# Characterization of gut contractility and microbiota in patients with severe chronic constipation

**DOI:** 10.1371/journal.pone.0235985

**Published:** 2020-07-17

**Authors:** Dina R. Yarullina, Marat U. Shafigullin, Kirill A. Sakulin, Anastasiia A. Arzamastseva, Ilnar F. Shaidullov, Maria I. Markelova, Tatiana V. Grigoryeva, Oleg Yu. Karpukhin, Guzel F. Sitdikova

**Affiliations:** 1 Department of Microbiology, Institute of Fundamental Medicine and Biology, Kazan Federal University, Kazan, Republic of Tatarstan, Russian Federation; 2 Department of Human and Animal Physiology, Institute of Fundamental Medicine and Biology, Kazan Federal University, Kazan, Republic of Tatarstan, Russian Federation; 3 Department of Surgical Diseases, Kazan State Medical University, Kazan, Republic of Tatarstan, Russian Federation; 4 "Omics Technologies" Laboratory, Institute of Fundamental Medicine and Biology, Kazan Federal University, Kazan, Republic of Tatarstan, Russian Federation; National Institute for Research on Agriculture, Alimentation and Environment (INRAE), FRANCE

## Abstract

Chronic constipation (CC) is one of the most common gastrointestinal disorders worldwide. Its pathogenesis, however, remains largely unclear. The purpose of the present work was to gain an insight into the role of contractility and microbiota in the etiology of CC. To this end, we studied spontaneous and evoked contractile activity of descending colon segments from patients that have undergone surgery for refractory forms of CC. The juxta-mucosal microbiota of these colon samples were characterized with culture-based and 16S rRNA sequencing techniques. In patients with CC the spontaneous colonic motility remained unchanged compared to the control group without dysfunction of intestinal motility. Moreover, contractions induced by potassium chloride and carbachol were increased in both circular and longitudinal colonic muscle strips, thus indicating preservation of contractile apparatus and increased sensitivity to cholinergic nerve stimulation in the constipated intestine. In the test group, the gut microbiota composition was assessed as being typically human, with four dominant bacterial phyla, namely *Firmicutes*, *Bacteroidetes*, *Proteobacteria*, and *Actinobacteria*, as well as usual representation of the most prevalent gut bacterial genera. Yet, significant inter-individual differences were revealed. The phylogenetic diversity of gut microbiota was not affected by age, sex, or colonic anatomy (dolichocolon, megacolon). The abundance of butyrate-producing genera *Roseburia*, *Coprococcus*, and *Faecalibacterium* was low, whereas conventional probiotic genera *Lactobacillus* and *Bifidobacteria* were not decreased in the gut microbiomes of the constipated patients. As evidenced by our study, specific microbial biomarkers for constipation state are absent. The results point to a probable role played by the overall gut microbiota at the functional level. To our knowledge, this is the first comprehensive characterization of CC pathogenesis, finding lack of disruption of motor activity of colonic smooth muscle cells and insufficiency of particular members of gut microbiota usually implicated in CC.

## Introduction

Chronic constipation (CC), also frequently referred to as functional constipation, colonic inertia, slow transit constipation, is a prevalent, distressing gastrointestinal disorder. Its etiology and pathophysiology remain poorly understood and is most likely multifactorial [1–3]. CC may develop as the result of sedentary lifestyle, the intake of harmful food, bad ecology, anomaly of colonic structure and its location in the abdominal cavity. Thus, constipation syndrome may be associated with dolichocolon, idiopathic megacolon, Payr’s syndrome, ptosis of the transverse colon, sigmocele, or may occur without anatomical abnormalities [4, 5]. The incidence rates of CC are higher in women than in men and they usually increase with age [6]. Since CС significantly reduces the patients’ life quality and can hardly be treated, the improvement of diagnostics and treatment of СС is one of the most important medical and social problems [7, 8]. Although conservative treatment of CC remains prevalent, surgical colonic resection has become more frequent nowadays, particularly in severe cases involving gut anatomical abnormality and dilatation, and has demonstrated long-term positive results [9–12].

Acquired and inherited anatomical and morphological changes in the colon are most frequently characterized by slow colonic transit and include a dilated or redundant colon like Hirschsprung’s disease, idiopathic megacolon, or dolichocolon [10, 13–15]. The etiology of idiopathic megacolon is not clear, and several alterations have been found in the morphology, functions, and regulation of intestinal motility [16]. Concerning smooth muscle function, decreases in frequency, amplitude, and duration of propulsive contractions of the colon have been reported in CC patients [17]. The evidence on the role of cholinergic innervation in the pathogenesis of CC is controversial. Some data have indicated a reduced activity of cholinergic nerves in patients with CC [18], thus suggesting a beneficial effect of cholinergic stimulation of distal colon [19, 20]. Another study has shown hypersensitivity of the colonic circular smooth muscle to cholinergic stimulation in CC patients [20]. Moreover, the administration of cholinesterase inhibitor did not improve the colonic motility in constipated patients [21]. It is still unclear whether constipation in a dilated or redundant colon is associated with impaired molecular mechanisms of contractility or neuronal regulation of smooth muscle cells.

The gut microbiota has been suggested to play a role in the pathogenesis of CC. End-products of fermentation and other bacterial metabolites may affect intestinal motor function through the mediators released by the gut immune response, or intestinal neuroendocrine factors [22]. Multiples studies have revealed differences in the composition of the intestinal microbiota in constipated patients compared with healthy controls, but these results are largely contradictory. For instance, in constipated obese children significant reduction in the representation of *Prevotella* and increased abundance of several genera of *Firmicutes*, including butyrate-producing *Coprococcus*, *Roseburia* and *Faecalibacterium*, were demonstrated based on 16S rRNA gene pyrosequencing [23]. Using culture-based techniques it was also shown that *Coprococcus*, *Roseburia* and *Faecalibacterium* tended to be increased in constipated patients [24, 25]. On the other hand, recent metagenomic study showed a reduced level of *Coprococcus 3*, *Roseburia* and *Bacteroides* and an increased level of *Faecalibacterium* in stool samples from individuals with functional constipation. No difference between constipated and control samples was indicated in regard *Prevotella* [26]. The inconsistency which exists in literature may possibly be explained by differences in resolution potential of culture-based and molecular approaches in assessment of the bulk of gut microbiota. The unculturable part of intestinal microbiota most likely was missed in culture-based investigations of stool samples collected from constipated individuals. Besides, studying of fecal microbiota instead of mucosal one results in a limited view of the gut microbiota. Although several microbial taxa were considered as promoting the symptoms of constipation [3, 23, 26], the causative agent of CC has not been established yet. It’s still questionable whether alterations in gut microbiota are a cause or a consequence of constipation. Indeed, dietary habits and slow colonic transit time may result in the altered gut microbiota in patients with constipation [27, 28].

The aim of the present study was to analyze the contractility and microbiota of colonic tissue samples from patients with refractory forms of CC.

## Material and methods

### Clinical characteristics of patients with CC

The study was approved by the Local Ethics Committees (Protocol No. 8, Kazan Federal University, 05.05.2015; Protocol No. 9, Kazan State Medical University, 24.11.2015). Written informed consent was obtained from all the patients enrolled in this study. The enrollment of participants and all the experiments complied with the relevant guidelines and institutional regulations. Data were collected from 20 patients (five males and fifteen females) who underwent colorectal surgery for refractory CC at the Republican Clinical Hospital of the Ministry of Health of the Republic of Tatarstan, between April 2015 and March 2018 (S1 Table). Their age ranged from 20 to 70 years, with an average age of 42 years (σ = ±13). Constipation syndrome in test group was identified in patients with dolichocolon (12 cases) (S1A Fig) and idiopathic megacolon (8 cases) (S1B Fig). Preoperative investigations included anamnesis, clinical examination, colonoscopy, colonic transit study by the rate of passage the radiopaque markers through the gastrointestinal tract, and computed tomography colonography for obtaining three-dimensional images of colons with abnormal architectonics and unusual location in the abdominal cavity. In patients with refractory CC in order to reduce the length of the colon the following types of surgery were performed: total, subtotal colectomy, left-sided hemicolectomy, and resection of the sigmoid colon. The descending colon segments were included in all resected specimens and were used to study intestinal motility and gut microbiota.

### Human tissue specimens and recording of contractile activity

The descending colon segments were transported to the laboratory in cold saline buffer. The contractile activity was analyzed according to previous studies [29, 30]. Longitudinal and circular strips (10 × 2 mm) were cut out from the tissue retaining all the layers of the intestinal wall. Colon specimens were suspended in a tissue chamber containing 10 mL Krebs solution (mM: NaCl, 121.0; KCl, 5.9; CaCl_2_, 2.5; MgCl_2_, 1.2; NaHCO_3_, 25.0; NaH_2_PO_4_, 1.2; glucose 8.0; bubbled with a mixture of 5% CO_2_/95% O_2_, pH 7.4) constantly bubbled with 5%CO_2_/95%O_2_ and the temperature was maintained at 37°C. One end of the specimen was fixed to a hook, and the other end was attached to an isometric force transducer (TSD125C0) connected to an amplifier to record the mechanical activity (Biopac Systems, USA). Data were digitalized (25 Hz) with AcqKnowledge 4.1 software (Biopac Systems, USA). The muscle strips were equilibrated for 60–90 min with a 1.5–2 g preload and 15-min bath washouts. After this, most of the strips displayed spontaneous phasic activity. A highly concentrated KCl solution (70 mM) was used to depolarize and stimulate the tissue, in which the concentration of NaCl was equivalently reduced to maintain the osmolarity. Carbachol, an agonist of acetylcholine receptors, was cumulatively added to the bath at increasing concentrations of 0.01, 0.1, 1, 10, 100, and 200 μM. The amplitudes of spontaneous and evoked contractile responses to KCl or carbachol were analyzed in both experimental groups.

The statistical analysis was performed using OriginPro (OriginLab Corp., USA) software. To normalize the data, the amplitude of the evoked response was divided by the weight of the tissue specimen. To analyze the dose-dependence curves, we used curve-fitting by the logistic function (y = A_2_ + (A_1_-A_2_) / (1 + (x / x_0_)^p^); A_1_ –initial value, A_2_ –final value, x0 –center (x_0_ = EC_50_) and “p” is the calculated power of sigmoidal curve) [31]. The difference between the data were estimated using Mann-Whitney test. The null hypothesis (H_0_) was set that there is no difference in contractile activity between the constipation group and the control group. The alternative hypothesis (H_A_) was that contractility is different in these two groups. A hypothesis test was declared statistical significance for a significance level of alpha = 0.05. Data are expressed as mean ± standard error of mean (M ± SEM), and “n” indicates the number of samples.

### Microbial culture methods for intestinal microbiota assessment

Fresh colonic samples were obtained from patients with CC (1 sample per patient; minimum 1 g) and were divided into two. One part was subjected to metagenomic sequencing to profile the entire community. The other part (0.5 g) was thoroughly washed with 10 mL of sterile physiological solution (0.85 g NaCl in 100 mL of milliQ water) for 30 min at 37°С and 180–200 rpm. The resulting suspension was serially diluted and plated onto different nutrient agar in Petri dishes as follows. A nonselective nutrient agar for the cultivation of microorganisms “BTN” (Biotechnovacia, Russia) was used as a plate count agar to assess total microbial growth. To distinguish between aerobic and facultative anaerobic bacteria, we applied pour or spread plate techniques and cultivation under aerobic or anaerobic conditions. Also, we used cabbage agar with 4% (w/v) CaCO_3_ to isolate lactic acid bacteria (LAB). *Lactobacillus* spp. were identified by using De Man, Rogosa, Sharpe (MRS) agar [32]. *Bifidobacterium* spp. were cultivated on Bifidobacterium agar (HiMedia Laboratories Limited, India). Bacteria of the family *Enterobacteriaceae* were inoculated onto Endo agar and SS agar (“Microgen”, Russia). Plates were incubated at 37 ^o^C for 72 h. To provide anaerobic conditions, we used “Anaerogas” gas-pak sachets (NIKI MLT, Russia). Bacterial colonies were identified based on their morphological properties.

### Metagenomic analyses

#### DNA extraction

Gut tissue samples were stored at –80°C before homogenization in a SuperFastPrep-1 (MP Biomedicals, USA) at 5 m/s for 2 min and DNA extraction with the Fast DNA SPIN Kit (MP Biomedicals, USA).

#### 16S rRNA gene amplification and sequencing

Sequences of the 16S rRNA gene were analyzed by next-generation sequencing using the MiSeq system (Illumina, USA). A 16S rRNA sequencing library was constructed according to the 16S Metagenomics Sequencing Library Preparation Protocol [33].

A first PCR round was performed with 4 μL of DNA extracted from microdissected tissues using primers targeting the 16S rRNA gene V3 and V4 regions, forward primer 341F (5′-*TCGTCGGCAGCGTCAGATGTGTATAAGAGACAGCC*TACGGGNGGCWGCAG-3′), and reverse primer 805R (5′*-GTCTCGTGGGCTCGGAGATGTGTATAAGAGACAG*GACTACHVGGGTATCTAATCC-3′), where Illumina adapters are indicated in italics [34]. Each 30 μL PCR reaction mix contained 2.0 μL (60–100 ng) template DNA, 1.5 μL of each primer (10 μM), 0.3 μL Q5 High Fidelity DNA polymerase (NEB, USA), 6 μL 5X Q5 Reaction Buffer, 0.6 μL dNTPs (10 mM), 18.1 μL dH_2_O. The PCR conditions for DNA amplification were as follows: initial denaturation at 98 ^o^C for 30 s, followed by 30 cycles of 98 ˚C denaturation for 10s, annealing at 55 ^o^C for 20 s and extension at 72 ^o^C for 30 s. After purification of PCR products with AMPure XT magnetic beads (Invitrogen), the second PCR was performed using primers from a Nextera XT Index Kit (Illumina). Each 50 μL PCR reaction mix contained 5.0 μL purified PCR-product, 5 μL of each primer (5 μM), 0.5 μL Q5 High Fidelity DNA polymerase (NEB, USA), 10 μL 5X Q5 Reaction Buffer, 1 μL dNTPs (10 mM), and 23.5 μL dH_2_O. The PCR conditions for DNA amplification were as follows: initial denaturation at 98 ^o^C for 30 s, followed by 8 cycles of 98 ^o^C denaturation for 15 s, annealing at 55 ^o^C for 30 s, and then extension at 72 ^o^C for 30 s. PCR-products purified with AMPure XT magnetic beads (Invitrogen) were visualized using gel electrophoresis and quantified with a Qubit dsDNA HS Assay Kit (Thermo Scientific) on a Qubit 2.0 fluorometer. The sample pool (4 nM) was denatured with 0.2 N NaOH, diluted further to 4 pM, and combined with 20% (v/v) denatured 4 pM PhiX, prepared following Illumina guidelines. Sequencing was performed with an Illumina MiSeq sequencer (Illumina, San Diego, CA, USA) using Illumina V3 chemistry and paired-end 2 × 300 base pair reads. All sequencing was performed in a single MiSeq run. Initial sequence data processing was performed by the Illumina MiSeq Reporter to demultiplex samples and remove the adapter. Primer sequence data were exported in FASTQ format. Sequencing was performed at the Interdisciplinary Center of Shared Facilities, Kazan Federal University [35].

#### Quantitative analysis of microbiome composition

Reads were processed and analyzed by phylogenetic and operational taxonomic unit (OTU) methods on QIIME software version 1.9.1 (http://qiime.org/) [36]. Paired-end reads were joined and then processed to remove low quality and chimeric sequence data. The rarefaction step was performed to reduce sequencing depth heterogeneity between samples. After this processing, sequences were clustered into OTUs at a sequence identity level of 97%. The latest GreenGenes database version 13.8 [37] was used.

To evaluate the alpha diversities of each microbiota community, we calculated phylogenetic distance metric [PD_whole_tree], Chao1, Shannon, and Simpson indices. Similarities between microbial compositions of samples were evaluated using the beta diversity characteristics, and both weighted and unweighted Unifrac were calculated on QIIME software. Principal coordinate analysis (PCoA) was performed to obtain the principal coordinates and visualize the species by using complex multidimensional data.

## Results

### Contractility of colon muscle strips from patients with CC

**Fig 1:**
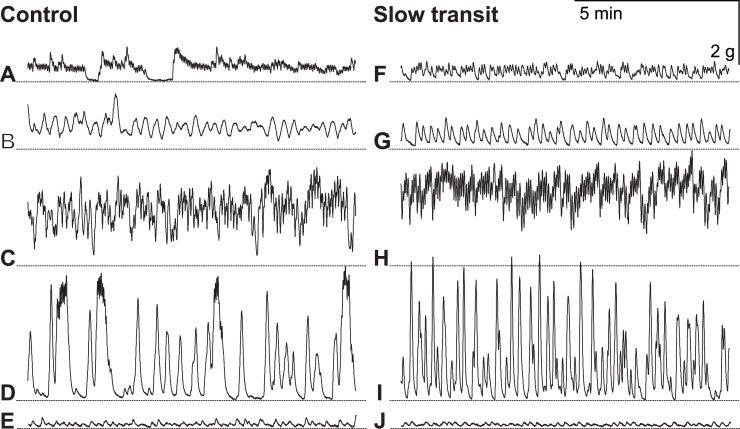
Spontaneous contractile activity of circular smooth muscle prepared from specimens of human descending colon from patients of the control group (**A, B, C, D, E**) and the slow transit (CC) group (**F, G, H, I, J**). The dashed line is set at the level of maximum relaxation.

In this study we compared colon muscle contractility of eight patients with refractory forms of CC (gender distribution: 2/6 (m/f), average age: 40 years (σ = ±12), range: 20–55 years) with that of a control group consisted of five patients with small (adeno)carcinoma of the sigmoid or rectosigmoid colon (T_2-3_N_0_M_0_) not associated with intestinal motility dysfunction (gender distribution: 2/3 (m/f), average age: 67 years (σ = ±5), range: 61–73 years) (S1 Table). Specimens obtained from patients with dolichocolon and idiopathic megacolon exhibited spontaneous activity of variable intensity. Spontaneous activity was not observed in 5 out of 10 longitudinal specimens from patients of the control group and in only 2 out of 16 specimens from patients with CC. Spontaneous activity was detected in all samples of circular fibers. Some specimens showed regular and irregular phasic contractions of different frequencies and amplitudes, while others demonstrated long phasic contractions without relaxation. Similar types of spontaneous activity were observed in smooth muscle strips of both groups. Examples of spontaneous activity of resected human colon specimens are shown in Fig 1.

**Fig 2:**
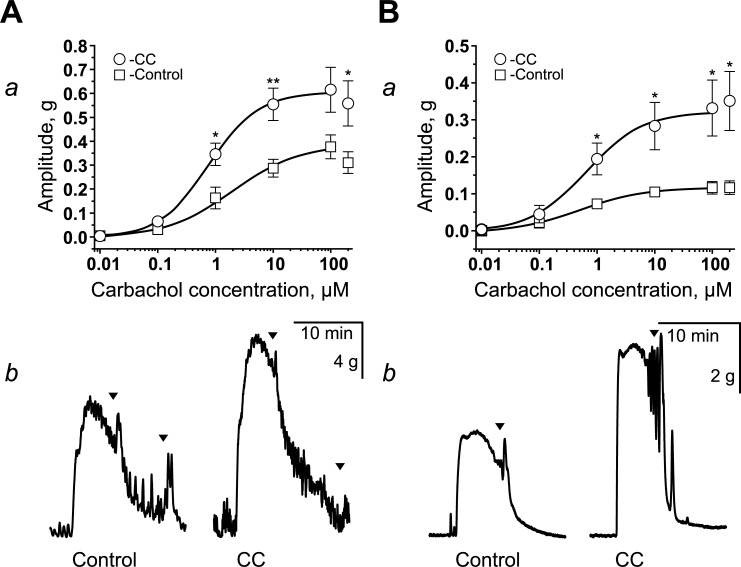
Carbachol-evoked contractions of descending colon strips from patients with CC (**A, B**). Dose-response effects of carbachol on the amplitude of contraction of circular (A**a**) and longitudinal (**Ba**) muscle strips in the control (white squares) and the CC groups (white circles). The amplitudes were normalized by the weight of specimens in each experiment. * p < 0.05; ** p < 0.01. Examples of contractions of circular (**Ab**) and longitudinal (**Bb**) muscle strips in the control and the CC groups in response to the application of carbachol (10 μM).

We used KCl (70 mM) or carbachol (0.01–200 μM) to assess the evoked contractility of circular and longitudinal smooth muscles (Fig 2). The average amplitudes of the contractions are shown in Table 1. Both the circular and longitudinal muscles of CC patients exhibited increased amplitude of contractions compared to the control group (Table 1). Carbachol induced a dose-dependent increase in the contractions of circular and longitudinal muscle specimens. However, the contractile responses of longitudinal strips were more variable (Fig 2A and 2B). In specimens from CC patients, contractions of higher amplitude were observed starting at 1 μM of carbachol. For the circular specimens, EC_50_ was 0.760 ± 0.063 μM in the CC group and 2.005 ± 1.046 μM in the control group (Fig 2A). For the longitudinal specimens, EC_50_ was 0.649 ± 0.141 μM in the CC group and 0.504 ± 0.047 μM (Fig 2B) in the control group.

**Table 1:** Amplitudes of KCl- and carbachol-evoked contractions of longitudinal and circular muscle specimens in the control (n = 5) and CC (n = 8) groups (mean ± SEM).

	Longitudinal		Circular	
	Control	CC	Control	CC
KCl	0.050±0.006	0.129±0.026 (p = 0.028)	0.073±0.014	0.192±0.033 (p = 0.028)
Carbachol 10 μM	0.105±0.014	0.283±0.064 (p = 0.014)	0.287±0.037	0.555±0.068 (p = 0.00988)

### Intra- and inter-individual variability of intestinal microbiota among constipated subjects

**Fig 3:**
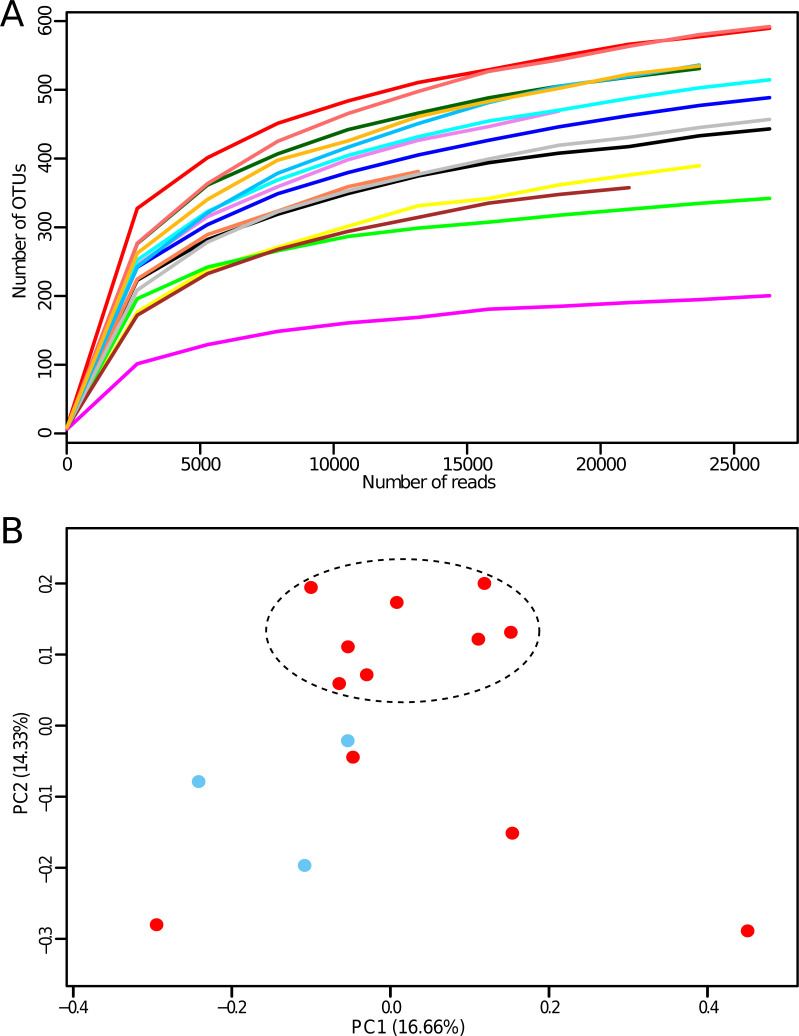
Evaluation of the alpha- (**A**) and beta-diversity (**B**). Panel **A** reports rarefaction curves based on the number of observed OTUs at increasing sequencing depth of the samples. Panel **B** shows the predicted PCoA. Datasets from men and women are colored in blue and red, respectively. Eight samples are grouped into a cluster, thus highlighting the similarity of their phylogenetic microbiota composition.

The study enrolled fifteen constipated patients with an average age of 42 years (σ = ±14, range: 20–70 years) and gender distribution 3/12 (m/f) (S1 Table). Colonic tissue samples were obtained during colectomy for CC to assess the microbiota composition by the conventional culture method and 16S rRNA-based sequencing analysis. The microbiological studies revealed significant inter-individual differences in the colonic mucosal microbiota of constipated patients (Table 2). MiSeq-mediated sequencing of the samples produced a total of 1,478,190 reads with an average of 98,546 ± 31,776 reads per sample (S2 Table). Raw sequence reads were deposited in NCBI Sequence Read Archive (SRA) under BioProject ID PRJNA590365 (https://www.ncbi.nlm.nih.gov/sra/PRJNA590365). Reads were further processed and analyzed using QIIME software, version 1.9.1 [36]. Joined paired-end reads were processed to remove low quality and chimeric sequence data. After quality filtering, chimera filtering, and rarefying, we analyzed 402,647 filtered reads, with an average of 26,843 ± 259 filtered reads per sample (S2 Table). Sequences were clustered into operational taxonomic units (OTU) based on the 97% identity threshold (open reference-based OTU picking strategy), the latest GreenGenes database version 13.8 [37] was used. Averages of 460 OTUs were identified across our dataset, with a minimum of 201 OTUs and a maximum of 592 OTUs. Alpha diversity indices were calculated using Chao1, Shannon, and Simpson metrics to characterize the richness and evenness of the bacterial community (S3 Table or Fig 3).

**Table 2:** Gut microbiota in patients with chronic constipation assessed by culture method.

Samples	Groups of microorganisms, lg CFU/g						
	Total bacterial growth of aerobic bacteria	Total bacterial growth of anaerobic bacteria	Lactic acid bacteria	*Lactobacillus* spp.	*Bifidobacterium* spp.	*Entero-bacteriaceae*	*Salmonella* spp., *Shigella* spp.
**1**	4±0.001	3±0.001	ND	ND	ND	ND	ND
**2**	2.85±0.21	1.24±0.34	5.92±4.35	Sporadic	ND	ND	ND
**3**	5.31±0.01	4.43±0.07	4.23±0.04	4.41±0.09	3.86±0.40	4.35±0.80	3.15±0.21
**4**	ND	ND	7±0.001	ND	ND	ND	ND
**5**	4.73±0.13	4.80±0.14	4.27±0.12	5.18±0.32	5.43±0.28	4.75±0.19	5.01±0.03
**7**	5.91±0.004	6.24±0.03	4.31±0.08	5.45±0.21	6.06±0.12	5.87±0.04	5.73±0.07
**8**	8.45±0.06	9.64±0.40	10.20±0.001	10.28±0.001	6.86±0.21	5.82±0.31	ND
**9**	3±0.001	ND	ND	ND	ND	ND	ND
**10**	ND	ND	ND	9±0.001	ND	ND	ND
**11**	Sporadic	ND	ND	ND	ND	ND	ND
**12**	15.13±0.07	14.00±0.03	14.21±0.02	13.03±0.03	14.22±0.02	15.93±0.75	ND
**13**	4.78±0.06	3.12±0.12	3.89±0.09	3.27±0.11	3.36±0.23	ND	ND
**14**	ND	ND	ND	ND	3±0.001	ND	ND
**15**	13.35±0.05	7.35±0.10	11.71±0.004	11.93±0.28	12.11±0.001	12.32±0.001	ND

Data are shown as mean ± SD. ND–not detected

The rarefaction curves we obtained showed that the retrieved sequencing data were adequate in all cases to cover the vast majority of biodiversity contained within the samples. Surprisingly, species diversity in colon tissue samples was lower than in stool samples, which are frequently used in similar studies of the gut microbiota of constipation.

To evaluate the inter-individual differences between gut microbiota of constipated patients, we assessed the beta-diversity characteristics [36], which were estimated using unweighted Unifrac measures [38] with further visualization through Principal Coordinate Analysis (PCoA). The PCoA plot showed that eight samples could be grouped into a cluster, thus highlighting the similarity of their phylogenetic microbiota composition. These eight samples belonged to women aged 20–53 years (Fig 3B). However, overall beta-diversity analyses did not reveal age- and sex-related clustering of the samples. Likewise, the microbiota composition of the samples was not associated with the type of colon pathology (dolichocolon or megacolon). Our results indicate that other genetic and environmental factors influence the phylogenetic diversity of the gut microbiomes during constipation.

### Taxonomic profiling of the gut microbiota of patients with CC

**Fig 4:**
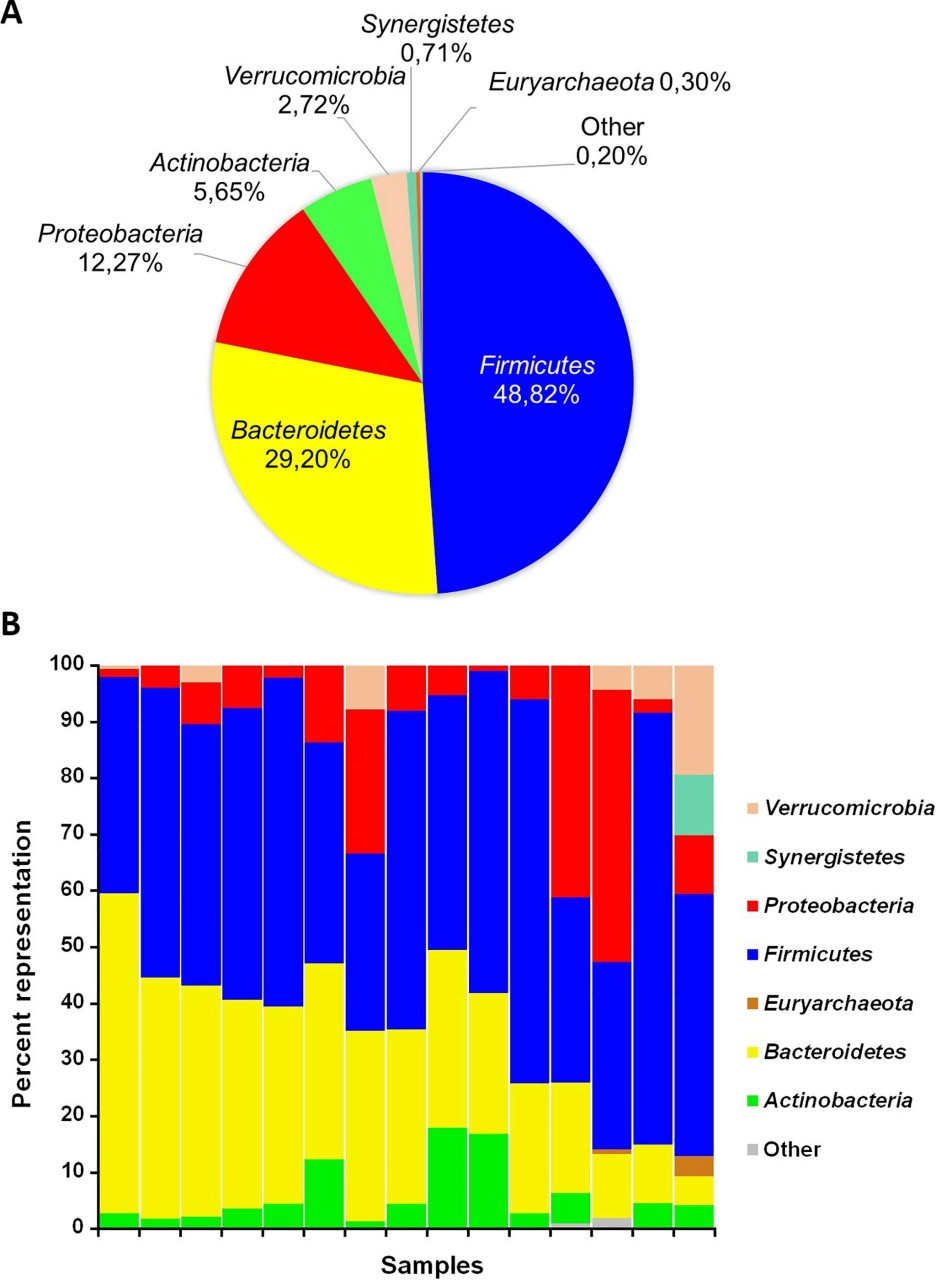
Phylum distribution in gut microbiomes of the constipated patients. (**A**) Average phylum distribution of the gut microbiomes of constipated patients; (**B**) phylum distribution of individual microbiomes.

We detected a total of 18 prokaryotic phyla in the gut microbiomes of constipated patients, including four frequently detected phyla, *Firmicutes*, *Bacteroidetes*, *Proteobacteria*, and *Actinobacteria*, and 14 minor phyla, *Euryarchaeota*, *Acidobacteria*, *Cyanobacteria*, *Elusimicrobia*, *Fusobacteria*, *Lentisphaerae*, *Planctomycetes*, *Spirochaetes*, *Synergistetes*, TM7, *Tenericutes*, *Verrucomicrobia*, WPS-2, and [*Thermi*] (Fig 4, S4 Table). The four frequently detected phyla were present in all samples, whereas each of the phyla *Acidobacteria*, *Elusimicrobia*, *Planctomycetes*, and *Spirochaetes*, was detected in only one sample. When analyzed at the genus level, the most frequently detected taxa in the samples were *Bacteroides* (18.0% ± 14.9%) along with unclassified members of the *Ruminococcaceae* (9.8% ± 7.3%), *Lachnospiraceae* (9.5% ± 6.9%), and *Enterobacteriaceae* families (8.0% ± 10.9%), and the *Prevotella* genus (5.5% ± 6.9%) (S5 Table).

We also compared the gut microbiota profiles obtained using culture methods (Table 2) with those obtained by 16S rDNA gene sequencing (S5 Table). As a result, we found discrepancies in the abundance of certain groups of bacteria, depending on the method used. Thus, five samples were tested negative for the 16S rRNA gene of *Lactobacillus* spp. but produced colonies when were inoculated on MRS agar, which is recommended for the cultivation of *Lactobacillus* spp. This may be attributed to the limited selectivity of the nutrient medium. In contrast, LAB, which were found in all tested samples by metagenomic studies of 16S rRNA, were not detected by culture-based technique in five samples. Similarly, bifidobacteria and enterobacteria, which were present according to the results of 16S rRNA gene sequencing, were not detected by culture method in five and eight samples, respectively. These sequences, revealed by genomic approaches, probably corresponded to uncultivated species and strictly anaerobic microorganisms that could not be detected by culture method.

## Discussion

CC is one of the most common gastrointestinal disorders. The majority of CC patients do not present organic pathology and are easily managed with medical treatment, including diet modification, exercise, and laxatives. However, there are severe forms associated with slow transit that are refractory to medical treatment. Idiopathic megacolon and dolichocolon are rare conditions that affect men and women equally and are associated with a permanently enlarged diameter or/and redundant colon [5, 15]. The pathogenesis of idiopathic megacolon is still unclear. Abnormalities of the enteric nervous system and alterations in the function of smooth muscle cells and connective tissue elements have been suggested to be involved in gut dysmotility [5, 13, 15]. Growing evidence indicates that intestinal microbiota may contribute to constipation and constipation-related symptoms [39]. However, the microbiota of patients with idiopathic megacolon and dolichocolon was not investigated. In this study, we analyzed the functional activity of smooth muscle cells and the gut microbiota of colonic tissue samples from constipated patients with megacolon and dolichocolon.

It is known that the contractile activity of the intestine is provided by longitudinal and circular smooth muscles whose contractions are coordinated by the central and enteric nervous systems, interstitial cells of Cajal, hormones, and autocrine and paracrine factors [40]. The contraction of smooth muscle cells is initiated by Ca^2+^ ions, activation of Ca-calmodulin dependent protein kinase with subsequent activation of myosin light chain kinase (MLCK) [40, 41]. In our study, the specimens obtained from patients with CC demonstrated spontaneous activity and the ability to contract in response to depolarization, which indicates that molecular mechanisms of the contractile activity were not impaired in the smooth muscle cells. Moreover, the maximum amplitudes of contractions in response to KCl application were larger compared to the control group.

One of the most important mechanisms that control the contractile activity of the human colon is the activation of the parasympathetic system and the release of acetylcholine, which binds to muscarinic receptors of types 2 and 3 (M_2_ and M_3_ ChRs) [42]. Although M_2_ ChRs prevails in the smooth muscle cells of the human colon, contractions in response to cholinomimetics are mainly mediated by M_3_ receptors. M_2_ receptors can enhance the contraction caused by the activation of M_3_ receptors [43], and their role increases in pathologies [44]. Previous studies with stimulation of colon strips from patients with CC demonstrated controversial results. Other researchers have observed a decrease [45] in contractile responses or a lack of difference in the amplitudes of carbachol-evoked contractions [46] in CC patients. We showed that EC_50_ for carbachol-evoked contractions was lower in the CC group, and the dose-response curves were shifted to the left, thus indicating an increased sensitivity to stimulating factors. Similarly to our results, it has been shown that smooth muscle cells in circular specimens of the sigmoid colon from patients with CC and diverticulosis exhibit an increased sensitivity to carbachol [20]. Hypersensitivity to cholinergic stimulation can be explained by the development of a secondary denervation syndrome, which is consistent with anomalies of intramural plexuses [47] observed in patients with CC and confirmed by functional cholinergic deficiency in response to electrical stimulation of intramural nerves [18]. Besides a reduced quantity of neurons, the number of neurons containing choline acetyltransferase decreased [48]. Increased expression of M_3_ ChRs in smooth muscle cells has also been observed in patients with diverticulosis [29], which might be a compensatory mechanism for reduced cholinergic innervation. At the same time, cholinesterase reduced colonic motor activity in patients with CC, whereas the inhibitor of choline esterase (edrofonium chloride) did not change the activity of the descending colon in patients with constipation [21]. These results could be explained by changes in the local environment of smooth muscle cells, atrophy of the mucous and muscular layers, inflammation, which impair the nervous regulation, sensitivity of contractile proteins, ion channels, and receptors [49, 50]. Also, the effects of exogenous or endogenous modulators able to inhibit the contractile activity of smooth muscle cells cannot be excluded. In particular, the intestinal microbiota may contribute to the pathogenesis of functional gastrointestinal disorders [39].

We used the culture method and 16S rRNA metagenomic analysis to study the gut microbiota in colonic tissue samples obtained from patients who had undergone colectomy for refractory forms of chronic constipation. Colonic tissue samples are seen as a direct approach, in contrast to fecal or mucosal samples, since they thoroughly replicate the profiles of the intestinal microbiota and provide thorough knowledge on the gut microbiome of patients with constipation.

Similar to previous descriptions [23, 26], we identified a preponderant presence of the genus *Bacteroides* in the gut of constipated patients (18.0% ± 14.9%). Being commensal, members of the genus *Bacteroides* account for about 25% of the total bacterial population in the adult human gut [51] and play a key role in the development and maintenance of intestinal sensory and motor functions [22]. For instance, *Bacteroides thetaiotaomicron* has been demonstrated to stimulate gut motility by increasing the expression of γ-aminobutyric, vesicle-associated protein-33, and enteric γ-actin [52]. In fact, according to 16S rDNA metagenomic profiling of fecal samples from healthy women, *Bacteroides* spp. were more abundant in loose stool of individuals with fast colon transit [53]. Concerning the role of *Bacteroides* in constipation, conflicting data have been found in the literature. There are reports on the depletion of the *Bacteroides* genus under functional constipation [26, 54, 55]. *Bacteroides fragilis* and *Bacteroides ovatus*, on the contrary, were selected as the most discriminative species in children with functional constipation, as opposed to healthy controls [56]. On the other side, Zhu *et al*. reported no differences in the *Bacteroides* genus content between constipated and healthy children [23]. In our study, 15.23% of reads belonging to the *Bacteroides* genus corresponded to unknown species, followed by *Bacteroides fragilis* (1.94%), and other species (*B*. *uniformis* (0.57%), *B*. *ovatus* (0.14%), *B*. *plebeius* (0.08%), *B*. *eggerthii* (0.02%), *B*. *caccae* (0.0036%), thus indicating that further genomic analyses are still needed to shed light on the biological role of this genus in constipation.

In previous studies, particular attention was given to butyrate-producing genera, such as *Coprococcus*, *Roseburia*, and *Faecalibacterium* [57]. Zhu *et al*. [23] reported that these taxa were more abundant in constipated obese children than in healthy controls. Conversely, Mancabelli *et al*. [26] demonstrated the depletion of *Roseburia* and *Coprococcus 3* in functionally constipated individuals, while *Faecalibacterium* content significantly increased. Butyrate was shown to produce opposite effects on gut motility: stimulation at low and inhibition at higher concentrations [58, 59]. Interestingly, fecal butyrate producers (i.e., *Faecalibacterium*, *Roseburia*, and *Coprococcus*) were associated with fast colonic transit [60], presumably because of induction of serotonin release and facilitating of cholinergic pathways by butyrate [61, 62]. On the other hand, mucosal butyrate producers (i.e. *Faecalibacterium*) were associated with constipation [60]. Several colonic effects of butyrate, such as inhibition of mucin secretion [63], reduction of stool volume [64] via stimulation of colonic water and electrolyte absorption [65], and inhibition of colonic smooth muscle contraction [59], could predispose to constipation. The butyrate-producing genera (*Roseburia*, 0.5% ± 0.008%; *Coprococcus*, 1.5% ± 0.019%; *Faecalibacterium*, 1.0% ± 0.014%) were present in our dataset as well, but in lower amounts compared to the data from previous metagenomic studies [23, 26]. Thus, chronic constipation in analyzed patients was not likely to be determined by the motility-inhibiting effect of butyrate-producing genera in the gut.

Previous metagenomic studies have identified 23 microbial taxa uniquely present in healthy individuals and absent in constipated subjects [26]. However, four of these genera, namely *Methanobrevibacter*, *Sedimentibacter*, *Leptotrichia*, and *Acinetobacter*, were found in our study. Thus, our results corroborate current opinion about the lack of specific microbial biomarkers whose presence or absence is associated with constipation and point at a probable role played by the overall gut microbiota at the functional level.

According to our data, *Akkermansia muciniphila* was in the top 10 most abundant species in the gut microbiota of the patients with chronic constipation (2.71%). Remarkably, the amount of *A*. *muciniphila* was highly variable: the taxon was not found in eight tested samples, while in seven samples, it varied from <1 (0.015)% up to 19.34%. *A*. *muciniphila* is an anaerobic Gram-negative mucin-degrading bacterium of the phylum *Verrucomicrobia* [66]. It usually inhabits the large intestine and accounts for approximately 3% of intestinal bacteria [67, 68]. The presence of *A*. *muciniphila* is associated with a healthy intestine, and its abundance is correlated to several disease states [66, 68–73], such as irritable bowel syndrome (IBS) [68, 71], acute appendicitis [72], obesity [70], and colorectal cancer [69, 73]. Although its role in human microbiome is still poorly understood, *A*. *muciniphila* is considered a beneficial microbe because of its effects on glucose and lipid metabolism and intestinal immunity [74]. To date, only a few studies have investigated the relationship between *Akkermansia* and chronic constipation. *Akkermansia* content increased in pseudo-germ-free mice after transplantation of fecal microbiota from patients with constipation. It was suggested that *Akkermansia* induced a depletion of the fecal water content due to degradation of intestinal mucin and, finally, impaired the intestinal mucosal barrier [75]. In this context, Vandeputte *et al*. [53] reported that *Akkermansia* populations increased with stool firmness and, thus, were more prevalent in slow transit individuals.

It is noteworthy that the abundance of common probiotic genera (*Bifidobacterium* and *Lactobacillus*) in the constipated patients did not decrease. *Bifidobacterium* spp. were detected in all analyzed samples, ranging from <0.1% up to 10.69%. Except for a small part of unclassified reads (0.22%), OTUs belonging to the *Bifidobacterium* genus were classified as *Bifidobacterium longum* (0.96%), *B*. *adolescentis* (0.36%), and *B*. *pseudolongum* (0.07%). Thus, the relative abundance and species representativity of bifidobacteria corresponded to those of healthy adults [76]. *Lactobacillus* spp. were observed in 11 out of 15 analyzed samples (73.3%) with a relative abundance of 0.1% ± 0.001%. So, their content did not differ significantly from that in healthy gut microbiota, where they normally constitute only a minor fraction, around 0.01 to 0.6% of total bacterial counts [77, 78]. Similarly, Zhu *et al*., using 16S rRNA gene pyrosequencing, demonstrated that the levels of *Lactobacillus* and *Bifidobacteria* species were not reduced in children with functional constipation [23]. Conversely, Khalif *et al*. [55] showed that the levels of *Bifidobacteria* and *Lactobacillus* significantly decreased in adult patients with constipation, perhaps, indicating a decrease in physiologically active representatives of these genera, because their conclusion was based on culture methods. Our results cast doubt on the efficacy of conventional probiotic treatment to restore the ecology of the gut microbiota in constipated patients.

## Conclusions

Thus, in this study we have demonstrated that the spontaneous motor activity of smooth muscle cells remains unchanged in patients with CC. Moreover, we detected an increased sensitivity to KCl- and carbachol-evoked contractions in circular and longitudinal muscle strips of the descending colon. Based on the previous data, we can suggest that smooth muscle cells develop a compensatory response to the abnormality in cholinergic stimulation.

Herein, we analyzed for the first time the gut microbiota composition of colon samples which thoroughly reflect the gut microbiota profiles of patients with refractory forms of CC. The results of this study suggest that the gut microbiome of constipated patients comprises typical gut bacteria along with significant inter-individual differences. Our data showed that these discrepancies were not affected by age, sex, and colonic anatomy (dolichocolon, megacolon). No definitive association was observed between constipation and the abundance or lack of certain prokaryotic taxa in the gut microbiome. Special attention was given to microbes that may affect motility via the production of metabolites. The butyrate-producing genera (*Roseburia*, *Coprococcus*, *Faecalibacterium*) were identified in low amount and, therefore, were not likely to promote motility-inhibiting activity. Our results showed that the abundances of common probiotic genera *Bifidobacterium* and *Lactobacillus* were not reduced, thus compromising the efficacy of conventional probiotic treatment to restore the ecology of the gut microbiota in constipated patients. Nevertheless, due to the limited number of samples, additional experiments are needed to validate these observations.

## Supporting information

S1 Fig Examples of abdominal computer tomography (CT) images in coronal section to illustrate the large colonic diameters. **A—**patient K., 23-year-old. Elongated transverse colon, sags in the small pelvis. Diagnosis: total dolichocolon, ptosis of the transverse colon; chronic constipation, stage of decompensation. **B**—patient M., 64-year-old. Total extension of the colon filled with fecal masses. Diagnosis: idiopathic megacolon; chronic constipation, stage of decompensation.
(TIF)

S1 Table Colonic samples collected in this study. (XLSX)

S2 Table 16S rRNA microbial profiling data. (XLSX)

S3 Table Alpha-diversity index and richness (number of OTUs) of samples. (XLSX)

S4 Table Taxonomic profiling of the samples at phylum level. (XLSX)

S5 Table Taxonomic profiling of the samples at genus level. (XLSX)

## References

[1] Bharucha AE, Dorn SD, Lembo A, Pressman A. American gastroenterological association medical position statement on constipation. Gastroenterology. 2013; 144:211–217. 10.1053/j.gastro.2012.10.029 23261064

[2] Lazebnik LB, Prilepskaia SI., Baryshnikov EN, Parfenov AI, Kosacheva TN. Prevalence and risk factors of constipation in the adult population of Moscow (according to population-based study MUSA). Eksp Klin Gastroenterol. 2011; 3:68–73. 21695954

[3] Zhao Y, Yu YB. Intestinal microbiota and chronic constipation. Springerplus. 2016; 5(1):1130 10.1186/s40064-016-2821-1 27478747 PMC4951383

[4] Wexner SD, Duthie GD. Constipation. Etiology, evaluation and management. 2nd ed Springer; 2006 10.1007/978-1-84628-275-1

[5] Cuda T, Gunnarsson R, de Costa A. Symptoms and diagnostic criteria of acquired Megacolon—a systematic literature review. BMC Gastroenterology. 2018; 18(1):25 10.1186/s12876-018-0753-7 29385992 PMC5793364

[6] Higgins PD, Johanson JF Epidemiology of constipation in North America: a systematic review. Am J Gastroenterol. 2004; 99(4):750–759. 10.1111/j.1572-0241.2004.04114.x 15089911

[7] Eoff JC, Lembo AJ. Optimal treatment of chronic constipation in managed care: review and roundtable discussion. J Manag Care Pharm. 2008; 14(9 Suppl. A):1–15. 10.18553/jmcp.2008.14.S8-A.1 18950252 PMC10438150

[8] Lacy BE, Levenick JM, Crowell M. Chronic constipation: new diagnostic and treatment approaches. Therap Adv Gastroenterol. 2012; 5(4):233–247. 10.1177/1756283X12443093 22778789 PMC3388525

[9] FitzHarris GP, Garcia-Aguilar J, Parker SC, Bullard KM, Madoff RD, Goldberg SM, et al Quality of life after subtotal colectomy for slow-transit constipation. Dis Colon Rectum. 2003; 46(4):433–440. 10.1007/s10350-004-6576-3 12682533

[10] Chatoor D, Emmnauel A. Constipation and evacuation disorders. Best Pract Res Clin Gastroenterol. 2009; 23(4):517–530. 10.1016/j.bpg.2009.05.001 19647687

[11] Dudekula A, Huftless S, Bielefeldt K. Colectomy for constipation: time trends and impact based on the US Nationwide Inpatient Sample, 1998–2011. Aliment Pharmacol Ther. 2015; 42(11–12):1281–1293. 10.1111/apt.13415 26423574

[12] Wei D. Progress in the treatment of surgical procedures for slow transit constipation. Zhonghua Wei Chang Wai Ke Za Zhi. 2018; 21(3):357–360. 29577225

[13] O'Dwyer RH, Acosta A, Camilleri M, Burton D, Busciglio I, Bharucha AE. Clinical features and colonic motor disturbances in chronic megacolon in adults. Dig Dis Sci. 2015; 60(8):2398–2407. 10.1007/s10620-015-3645-5 25868630 PMC4499849

[14] Raahave D. Dolichocolon revisited: an inborn anatomic variant with redundancies causing constipation and volvulus. World J Gastrointest Surg. 2018; 10(2):6–12. 10.4240/wjgs.v10.i2.6 29492185 PMC5827035

[15] Wang XJ, Camilleri M. Chronic megacolon presenting in adolescents or adults: clinical manifestations, diagnosis, and genetic associations. Dig Dis Sci. 2019; 64(10):2750–2756. 10.1007/s10620-019-05605-7 30953226 PMC6744965

[16] Kellow JE, Azpiroz F, Delvaux M, Gebhart GF, Mertz HR, Quigley EM, et al Applied principles of neurogastroenterology: physiology/motility sensation. Gastroenterology. 2006; 130(5):1412–1420. 10.1053/j.gastro.2005.08.061 16678555

[17] Bassotti G, Chiarioni G, Vantini I, Betti C, Fusaro C, Pelli MA, et al Anorectal manometric abnormalities and colonic propulsive impairment in patients with severe chronic idiopathic constipation. Dig Dis Sci. 1994; 39(7):1558–1564. 10.1007/BF02088064 8026270

[18] Burleigh DB. Evidence for a functional cholinergic deficit in human colonic tissue resected for constipation. J. Pharm. Pharmacol. 1988; 40:55–57. 10.1111/j.2042-7158.1988.tb05151.x 2896776

[19] Reynolds JC, Ouyang A, Lee CA, Baker L, Sunshine AG, Cohen S. Chronic severe constipation. Prospective motility studies in 25 consecutive patients. Gastroenterology. 1987; 92(2):414–420. 3792778

[20] Slater BJ, Varma JS, Gillespie JI. Abnormalities in the contractile properties of colonic smooth muscle in idiopathic slow transit constipation. Br J Surg. 1997; 84:181–184. 9052428

[21] Bassotti G, Chiarioni G, Imbimbo BP, Betti C, Bonfante F, Vantini I, et al Impaired colonic motor response to cholinergic stimulation in patients with severe chronic idiopathic (slow transit type) constipation. Dig Dis Sci. 1993; 38(6):1040–1045. 10.1007/BF01295719 8508698

[22] Barbara G, Stanghellini V, Brandi G, Cremon C, Di Nardo G, De Giorgio R, et al Interactions between commensal bacteria and gut sensorimotor function in health and disease. Am J Gastroenterol. 2005; 100(11):2560–2568. 10.1111/j.1572-0241.2005.00230.x 16279914

[23] Zhu L, Liu W, Alkhouri R, Baker RD, Bard JE, Quigley EM, et al Structural changes in the gut microbiome of constipated patients. Physiol Genomics. 2014; 46(18):679–686. 10.1152/physiolgenomics.00082.2014 25073603

[24] Pryde SE, Duncan SH, Hold GL, Stewart CS, Flint HJ. The microbiology of butyrate formation in the human colon. FEMS Microbiol. Lett. 2002; 217(2):133–139. 10.1111/j.1574-6968.2002.tb11467.x 12480096

[25] Sokol H, Pigneur B, Watterlot L, Lakhdari O, Bermúdez-Humarán LG, Gratadoux JJ, et al *Faecalibacterium prausnitzii* is an anti-inflammatory commensal bacterium identified by gut microbiota analysis of Crohn disease patients. Proc Natl Acad Sci U S A. 2008; 105(43):16731–16736. 10.1073/pnas.0804812105 18936492 PMC2575488

[26] Mancabelli L, Milani C, Lugli GA, Turroni F, Mangifesta M, Viappiani A, et al Unveiling the gut microbiota composition and functionality associated with constipation through metagenomic analyses. Sci Rep. 2017; 7(1):9879 10.1038/s41598-017-10663-w 28852182 PMC5575163

[27] Kashyap PC, Marcobal A, Ursell LK, Larauche M, Duboc H, Earle KA, et al Complex interactions among diet, gastrointestinal transit, and gut microbiota in humanized mice. Gastroenterology. 2013; 144(5):967–977. 10.1053/j.gastro.2013.01.047 23380084 PMC3890323

[28] Dimidi E, Christodoulides S, Scott SM, Whelan K. Mechanisms of action of probiotics and the gastrointestinal microbiota on gut motility and constipation. Adv Nutr. 2017; 8(3):484–494. 10.3945/an.116.014407 28507013 PMC5421123

[29] Golder M, Burleigh DE, Belai A, Ghali L, Ashby D, Lunniss PJ, et al Smooth muscle cholinergic denervation hypersensitivity in diverticular disease. Lancet. 2003; 361(9373):1945–1951. 10.1016/S0140-6736(03)13583-0 12801738

[30] Gabitova DM, Shaidullov IF, Sabirullina GI, Shafigullin MU, Sitdikov FG, Sitdikova GF. Role of cyclic nucleotides in the effect of hydrogen sulfide on contractions of rat jejunum. Bull Exp Biol Med. 2017; 163(1):14–17. 10.1007/s10517-017-3726-x 28580487

[31] Hall DA, Langmead CJ. Matching models to data: a receptor pharmacologist's guide. Br J Pharmacol. 2010; 161(6):1276–1290. 10.1111/j.1476-5381.2010.00879.x 20977467 PMC3000653

[32] De Man JC, Rogosa M, Sharpe EM. A medium for the cultivation of lactobacilli. J Appl Bacteriol. 1960; 23(1):130–135. 10.1111/j.1365-2672.1960.tb00188.x

[33] 16S metagenomic sequencing library preparation. Illumina, San Diego, CA; 2013. Available from: https://web.uri.edu/gsc/files/16s-metagenomic-library-prep-guide-15044223-b.pdf

[34] Saffarian A, Mulet C, Regnault B, Amiot A, Tran-Van-Nhieu J, Ravel J, et al Crypt- and mucosa-associated core microbiotas in humans and their alteration in colon cancer patients. mBio. 2019; 10(4):e01315–01319. 10.1128/mBio.01315-19 31311881 PMC6635529

[35] Watt E, Gemmell MR, Berry S, Glaire M, Farquharson F, Louis P, et al Extending colonic mucosal microbiome analysis-assessment of colonic lavage as a proxy for endoscopic colonic biopsies. Microbiome. 2016; 4(1):61 10.1186/s40168-016-0207-9 27884202 PMC5123352

[36] Caporaso JG, Kuczynski J, Stombaugh J, Bittinger K, Bushman FD, Costello EK, et al QIIME allows analysis of high-throughput community sequencing data. Nat Methods. 2010; 7(5):335–336. 10.1038/nmeth.f.303 20383131 PMC3156573

[37] DeSantis TZ, Hugenholtz P, Larsen N, Rojas M, Brodie EL, Keller K, et al Greengenes, a chimera-checked 16S rRNA gene database and workbench compatible with ARB. Appl Environ Microbiol. 2006; 72(7):5069–5072. 10.1128/AEM.03006-05 16820507 PMC1489311

[38] Lozupone C, Lladser ME, Knights D, Stombaugh J, Knight R. UniFrac: an effective distance metric for microbial community comparison. ISME J. 2011; 5(2):169–172. 10.1038/ismej.2010.133 20827291 PMC3105689

[39] Ge X, Zhao W, Ding C, Tian H, Xu L, Wang H, et al Potential role of fecal microbiota from patients with slow transit constipation in the regulation of gastrointestinal motility. Sci Rep. 2017; 7(1):441 10.1038/s41598-017-00612-y 28348415 PMC5428802

[40] Sanders KM, Koh SD, Ro S, Ward SM. Regulation of gastrointestinal motility -insights from smooth muscle biology. Nat Rev Gastroenterol Hepatol. 2012; 9(11):633–645. 10.1038/nrgastro.2012.168 22965426 PMC4793911

[41] Boyer JC, Magous R, Christen MO, Balmes JL, Bali JP. Contraction of human colonic circular smooth muscle cells is inhibited by the calcium сhannel blocker pinaverium bromide. Cell Calcium. 2001; 29(6):429–438. 10.1054/ceca.2001.0205 11352508

[42] Gómez A, Martos F, Bellido I, Marquez E, Garcia AJ, Pavia J, et al Muscarinic receptor subtypes in human and rat colon smooth muscle. Biochem Pharmacol. 1992 43(11):2413–2419. 10.1016/0006-2952(92)90321-9 1610405

[43] Mansfield KJ, Mitchelson FJ, Moore KH, Burcher E. Muscarinic receptor subtypes in the human colon: lack of evidence for atypical subtypes. Eur J Pharmacol. 2003; 482(1–3):101–109. 10.1016/j.ejphar.2003.10.008 14660010

[44] Uchiyama T, Chess-Williams R. Muscarinic receptor subtypes of the bladder and gastrointestinal tract. J Smooth Muscle Res. 2004; 40(6):237–247. 10.1540/jsmr.40.237 15725706

[45] Tomita R, Tanjoh K, Fujisaki S, Ikeda T, Fukuzawa M. Regulation of the enteric nervous system in the colon of patients with slow transit constipation. Hepatogastroenterology. 2002; 49(48):1540–1544. 12397730

[46] Menzies JR, McKee R, Corbett AD. Differential alterations in tachykinin NK2 receptors in isolated colonic circular smooth muscle in inflammatory bowel disease and idiopathic chronic constipation. Regul Pept. 2001; 99(2–3):151–156. 10.1016/s0167-0115(01)00244-0 11384776

[47] Krishnamurthy S, Schuffler MD, Rohrmann CA, Pope CE 2nd. Severe idiopathic constipation is associated with a distinctive abnormality of the colonic myenteric plexus. Gastroenterology. 1985; 88:26–34. 10.1016/s0016-5085(85)80128-1 3964770

[48] Wattchow D, Brookes S, Murphy E, Carbone S, de Fontgalland D, Costa M. Regional variation in the neurochemical coding of the myenteric plexus of the human colon and changes in patients with slow transit constipation. Neurogastroenterol Motil. 2008; 20(12):1298–1305. 10.1111/j.1365-2982.2008.01165.x 18662329

[49] Jarrett ME, Mowatt G, Glazener CM, Fraser C, Nicholls RJ, Grant AM, et al Systematic review of sacral nerve stimulation for faecal incontinence and constipation. Br J Surg. 2004; 91(12):1559–1569. 10.1002/bjs.4796 15455360

[50] Ohama T, Hori M, Ozaki H. Mechanism of abnormal intestinal motility in inflammatory bowel disease: how smooth muscle contraction is reduced? J Smooth Muscle Res. 2007; 43(2):43–54. 10.1540/jsmr.43.43 17598957

[51] Moore WE, Cato EP, Holdeman LV. Some current concepts in intestinal bacteriology. Am J Clin Nutr. 1978; 31(10 S):33–42. 10.1093/ajcn/31.10.S33 707392

[52] Hooper LV, Wong MH, Thelin A, Hansson L, Falk PG, Gordon JI. Molecular analysis of commensal host-microbial relationships in the intestine. Science. 2001; 291(5505):881–884. 10.1126/science.291.5505.881 11157169

[53] Vandeputte D, Falony G, Vieira-Silva S, Tito RY, Joossens M, Raes J. Stool consistency is strongly associated with gut microbiota richness and composition, enterotypes and bacterial growth rates. Gut. 2016; 65(1):57–62. 10.1136/gutjnl-2015-309618 26069274 PMC4717365

[54] Kim SE, Choi SC, Park KS, Park MI, Shin JE, Lee TH, et al Change of fecal flora and effectiveness of the short-term VSL#3 Probiotic treatment in patients with functional constipation. J Neurogastroenterol Motil. 2015; 21(1):111–120. 10.5056/jnm14048 25537674 PMC4288088

[55] Khalif IL, Quigley EM, Konovitch EA, Maximova ID. Alterations in the colonic flora and intestinal permeability and evidence of immune activation in chronic constipation. Dig Liver Dis. 2005; 37(11):838–849. 10.1016/j.dld.2005.06.008 16169298

[56] de Meij TG, de Groot EF, Eck A, Budding AE, Kneepkens CM, Benninga MA, et al Characterization of microbiota in children with chronic functional constipation. PLoS One. 2016; 11(10):e0164731 10.1371/journal.pone.0164731 27760208 PMC5070844

[57] Louis P, Flint HJ. Diversity, metabolism and microbial ecology of butyrate-producing bacteria from the human large intestine. FEMS Microbiol. Lett. 2009; 294(1):1–8. 10.1111/j.1574-6968.2009.01514.x 19222573

[58] Neunlist M, Dobreva G, Schemann M. Characteristics of mucosally projecting myenteric neurones in the guinea-pig proximal colon. J Physiol. 1999; 517(Pt 2):533–546. 10.1111/j.1469-7793.1999.0533t.x 10332100 PMC2269343

[59] Squires PE, Rumsey RD, Edwards CA, Read NW. Effect of short-chain fatty acids on contractile activity and fluid flow in rat colon *in vitro*. Am J Physiol. 1992; 262(5 Pt 1):813–817. 10.1152/ajpgi.1992.262.5.G813 1590391

[60] Parthasarathy G, Chen J, Chen X, Chia N, O'Connor HM, Wolf PG, et al Relationship between microbiota of the colonic mucosa vs feces and symptoms, colonic transit, and methane production in female patients with chronic constipation. Gastroenterology. 2016; 150(2):367–379.e1. 10.1053/j.gastro.2015.10.005 26460205 PMC4727996

[61] Reigstad CS, Salmonson CE, Rainey JF 3rd, Szurszewski JH, Linden DR, Sonnenburg JL, et al Gut microbes promote colonic serotonin production through an effect of short-chain fatty acids on enterochromaffin cells. FASEB J. 2015; 29(4):1395–1403. 10.1096/fj.14-259598 25550456 PMC4396604

[62] Soret R, Chevalier J, De Coppet P, Poupeau G, Derkinderen P, Segain JP, et al Short-chain fatty acids regulate the enteric neurons and control gastrointestinal motility in rats. Gastroenterology. 2010; 138(5):1772–1782. 10.1053/j.gastro.2010.01.053 20152836

[63] Barcelo A, Claustre J, Moro F, Chayvialle JA, Cuber JC, Plaisancié P. Mucin secretion is modulated by luminal factors in the isolated vascularly perfused rat colon. Gut. 2000; 46(2):218–224. 10.1136/gut.46.2.218 10644316 PMC1727811

[64] Canani RB, Terrin G, Cirillo P, Castaldo G, Salvatore F, Cardillo G, et al Butyrate as an effective treatment of congenital chloride diarrhea. Gastroenterology. 2004; 127(2):630–634. 10.1053/j.gastro.2004.03.071 15300594

[65] Binder HJ, Mehta P. Short-chain fatty acids stimulate active sodium and chloride absorption *in vitro* in the rat distal colon. Gastroenterology. 1989; 96:989–996. 10.1016/0016-5085(89)91614-4 2925072

[66] Geerlings SY, Kostopoulos I, de Vos WM, Belzer C. *Akkermansia muciniphila* in the human gastrointestinal tract: when, where, and how? Microorganisms. 2018; 6(3):75 10.3390/microorganisms6030075 30041463 PMC6163243

[67] Belzer C, de Vos WM. Microbes inside—from diversity to function: the case of *Akkermansia*. ISME J. 2012; 6(8):1449–1458. 10.1038/ismej.2012.6 22437156 PMC3401025

[68] Png CW, Lindén SK, Gilshenan KS, Zoetendal EG, McSweeney CS, Sly LI, et al Mucolytic bacteria with increased prevalence in IBD mucosa augment *in vitro* utilization of mucin by other bacteria. Am J Gastroenterol. 2010; 105(11):2420–2428. 10.1038/ajg.2010.281 20648002

[69] Dingemanse C, Belzer C, van Hijum SA, Günthel M, Salvatori D, den Dunnen JT, et al *Akkermansia muciniphila* and *Helicobacter typhlonius* modulate intestinal tumor development in mice. Carcinogenesis. 2015; 36(11):1388–1396. 10.1093/carcin/bgv120 26320104

[70] Karlsson CL, Onnerfält J, Xu J, Molin G, Ahrné S, Thorngren-Jerneck K. The microbiota of the gut in preschool children with normal and excessive body weight. Obesity (Silver Spring). 2012; 20(11):2257–2261. 10.1038/oby.2012.110 22546742

[71] Rajilic-Stojanovic M, Shanahan F, Guarner F, de Vos WM. Phylogenetic analysis of dysbiosis in ulcerative colitis during remission. Inflamm. Bowel Dis. 2013; 19(3):481–488. 10.1097/MIB.0b013e31827fec6d 23385241

[72] Swidsinski A, Dörffel Y, Loening-Baucke V, Theissig F, Rückert JC, Ismail M, et al Acute appendicitis is characterised by local invasion with *Fusobacterium nucleatum/necrophorum*. Gut. 2011; 60(1):34–40. 10.1136/gut.2009.191320 19926616

[73] Weitman ES, Aschen SZ, Farias-Eisner G, Albano N, Cuzzone DA, Ghanta S, et al Obesity impairs lymphatic fluid transport and dendritic cell migration to lymph nodes. PLoS One. 2013; 8(8):e70703 10.1371/journal.pone.0070703 23950984 PMC3741281

[74] Naito Y, Uchiyama K, Takagim T. A next-generation beneficial microbe: *Akkermansia muciniphila*. J. Clin Biochem Nutr. 2018; 63(1):33–35. 10.3164/jcbn.18-57 30087541 PMC6064808

[75] Cao H, Liu X, An Y, Zhou G, Liu Y, Xu M, et al Dysbiosis contributes to chronic constipation development via regulation of serotonin transporter in the intestine. Sci Rep. 2017; 7(1):10322 10.1038/s41598-017-10835-8 28871143 PMC5583244

[76] Arboleya S, Watkins C, Stanton C, Ross RP. Gut bifidobacteria populations in human health and aging. Front Microbiol. 2016; 7:1204 10.3389/fmicb.2016.01204 27594848 PMC4990546

[77] Harmsen HJ, Raangs GC, He T, Degener JE, Welling GW. Extensive set of 16S rRNA-based probes for detection of bacteria in human feces. Appl Environ Microbiol. 2002; 68(6):2982–90. 10.1128/aem.68.6.2982-2990.2002 12039758 PMC123985

[78] Ilinskaya ON, Ulyanova VV, Yarullina DR, Gataullin IG. Secretome of intestinal bacilli: a natural guard against pathologies. Front Microbiol. 2017; 8:1666 10.3389/fmicb.2017.01666 28919884 PMC5586196

