# Supplementary figures and images for: Characterization of gut contractility and microbiota in patients with severe chronic constipation
Source: PLoS One. 2020 Jul 17;15(7):e0235985. doi: 10.1371/journal.pone.0235985 (PMC7367488; doi:10.1371/journal.pone.0235985)

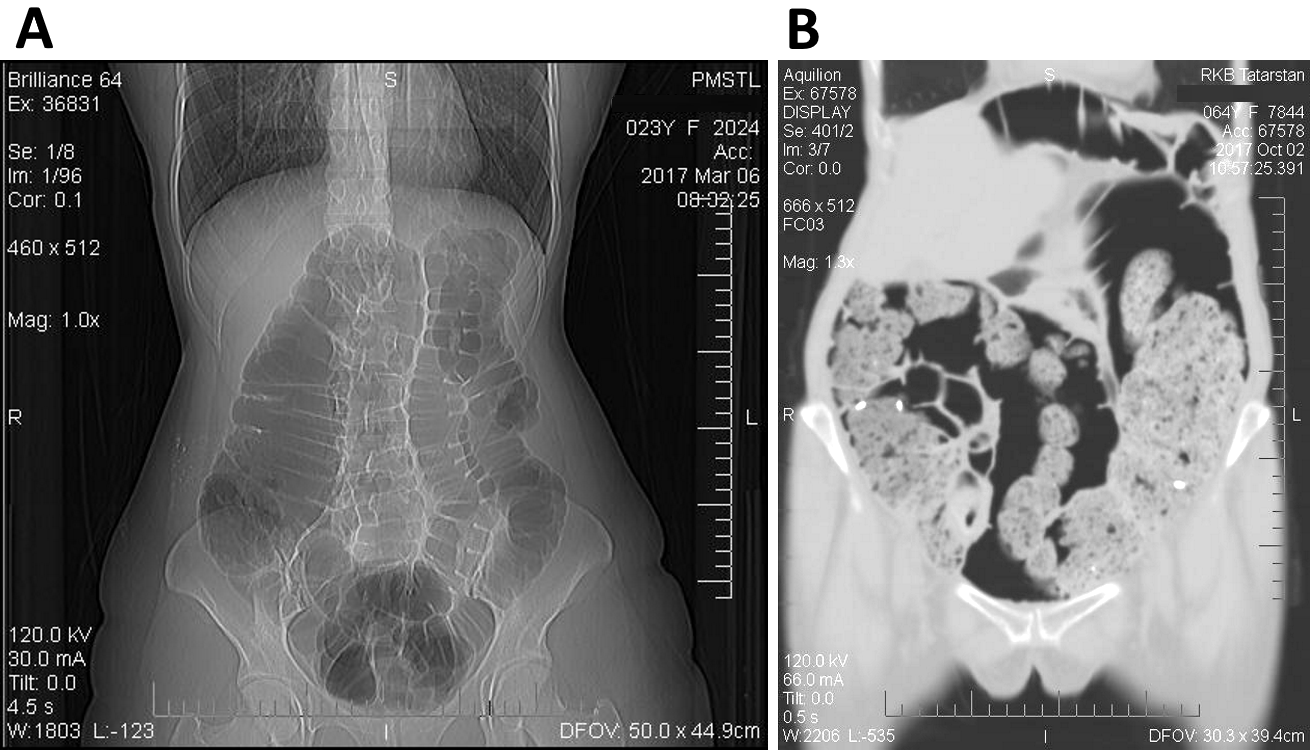

Supplement: S1 Fig — A—patient K., 23-year-old. Elongated transverse colon, sags in the small pelvis. Diagnosis: total dolichocolon, ptosis of the transverse colon; chronic constipation, stage of decompensation. B—patient M., 64-year-old. Total extension of the colon filled with fecal masses. Diagnosis: idiopathic megacolon; chronic constipation, stage of decompensation. (TIF) [file pone.0235985.s001.tif]
